# Supplementary material for: Subjective socioeconomic status moderates depression’s impact on fairness perception in the ultimatum game: A moderated mediation model
Source: PLoS One. 2025 Sep 2;20(9):e0330870. doi: 10.1371/journal.pone.0330870 (PMC12404385; doi:10.1371/journal.pone.0330870)
Supplement: S1 Appendix — (DOCX) [file pone.0330870.s001.docx]

Welcome, and thank you for participating in this study. In this questionnaire, you will receive a series of offers from others to split the money. You can choose to accept or reject the offer.

If you **accept**, you and the person will receive the proposed amounts.

If you **reject**, you and the person will receive nothing.

The proposers are from a series of anonymous students. After you make your choice, I will give them the corresponding money according to your choice.

Please consider your choice carefully:

|  |  |  |  |
| --- | --- | --- | --- |
| Proposer number | Proposer gets (Cent) | You get (Cent) | If you think it is ok,  please tick **√**, if not, please tick **×** |
| 936 | **50** | **50** |  |
| 357 | **70** | **30** |  |
| 118 | **90** | **10** |  |
| 330 | **30** | **70** |  |
| 267 | **10** | **90** |  |
| 630 | **30** | **70** |  |
| 109 | **20** | **80** |  |
| 531 | **80** | **20** |  |
| 221 | **60** | **40** |  |
| 633 | **90** | **10** |  |
| 377 | **70** | **30** |  |
| 314 | **10** | **90** |  |
| 256 | **60** | **40** |  |
| 127 | **80** | **20** |  |
| 904 | **50** | **50** |  |
| 812 | **40** | **60** |  |
| 513 | **40** | **60** |  |
| 101 | **20** | **80** |  |

**Please describe your sense of fairness of different proposals on a 7-point scale, with 1 being very unfair and 7 being very fair.**

| **Proposer gets** | **You**  **get** | Very unfair | unfair | Somewhat Unfair | Neither Fair Nor Unfair | Somewhat  Fair | Fair | Very Fair |
| --- | --- | --- | --- | --- | --- | --- | --- | --- |
| **50** | **50** |  |  |  |  |  |  |  |
| **10** | **90** |  |  |  |  |  |  |  |
| **30** | **70** |  |  |  |  |  |  |  |
| **40** | **60** |  |  |  |  |  |  |  |
| **90** | **10** |  |  |  |  |  |  |  |
| **80** | **20** |  |  |  |  |  |  |  |
| **20** | **80** |  |  |  |  |  |  |  |
| **70** | **30** |  |  |  |  |  |  |  |
| **60** | **40** |  |  |  |  |  |  |  |

Below is a list of the ways you might have felt or behaved. Please tell me how often you have felt this way during the past week.

1 = Rarely or None of the Time (Less than 1 Day)

2 = Some or a Little of the Time (1-2 Days)

3 = Occasionally or a Moderate Amount of Time (3-4 Days)

4 = Most or All of the Time (5-7 Days)

**During the past week:**

|  | **Rarely or none of the time**  (less than 1 day) | **Some or a little of the time**  (1‐2 days) | **Occasionally or a moderate amount of time**  (3‐4 days) | **Most or all of the time**  (5‐7 days) |
| --- | --- | --- | --- | --- |
| 1. I was bothered by things that  usually don't bother me. | □ | □ | □ | □ |
| 2. I did not feel like eating; my  appetite was poor. | □ | □ | □ | □ |
| 3. I felt that I could not shake  off the blues even with help from my family or friends. | □ | □ | □ | □ |
| 4. I felt that I was just as good  as other people. | □ | □ | □ | □ |
| 5. I had trouble keeping my  mind on what I was doing. | □ | □ | □ | □ |
| 6. I felt depressed. | □ | □ | □ | □ |
| 7. I felt that everything I did  was an effort. | □ | □ | □ | □ |
| 8. I felt hopeful about the  future. | □ | □ | □ | □ |
| 9. I thought my life had been a  failure. | □ | □ | □ | □ |
| 10. I felt fearful. | □ | □ | □ | □ |
| 11. My sleep was restless. | □ | □ | □ | □ |
| 12. I was happy. | □ | □ | □ | □ |
| 13. I talked less than usual. | □ | □ | □ | □ |
| 14. I felt lonely. | □ | □ | □ | □ |
| 15. People were unfriendly. | □ | □ | □ | □ |
| 16. I enjoyed life. | □ | □ | □ | □ |
| 17. I had crying spells. | □ | □ | □ | □ |
| 18. I felt sad. | □ | □ | □ | □ |
| 19. I felt that people dislike me. | □ | □ | □ | □ |
| 20. I could not get “going”. | □ | □ | □ | □ |

The top of the ladder (10) represents people at the top of society, and the bottom of the ladder (1) represents people at the bottom of society.

**Please mark the position on the ladder where you think you or your family are.**


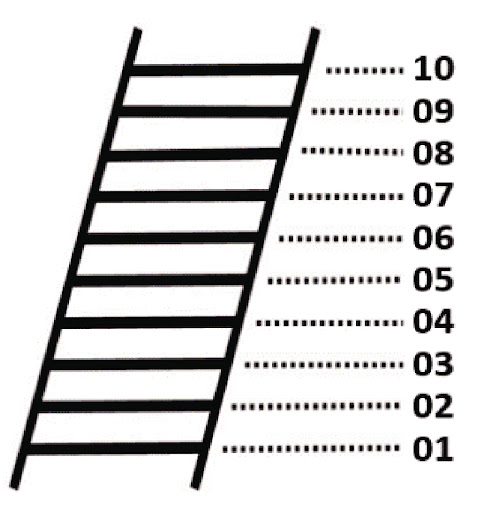


Name:

Age:

Gender:

Ethnicity:

Location:

Education Background:

Language:

Religion:

Annual Household Income:
